# Creation of an Isogenic Human iPSC-Based RGC Model of Dominant Optic Atrophy Harboring the Pathogenic Variant c.1861C>T (p.Gln621Ter) in the *OPA1* Gene

**DOI:** 10.3390/ijms25137240

**Published:** 2024-06-30

**Authors:** Marta García-López, Lydia Jiménez-Vicente, Raquel González-Jabardo, Helena Dorado, Irene Gómez-Manjón, Miguel Ángel Martín, Carmen Ayuso, Joaquín Arenas, María Esther Gallardo

**Affiliations:** 1 Grupo de Investigación Traslacional con Células iPS, Instituto de Investigación Sanitaria Hospital 12 de Octubre (imas12), 28041 Madrid, Spain; 2 Servicio de Genética, Hospital 12 de Octubre, Instituto de Investigación Sanitaria Hospital 12 de Octubre (imas12), 28041 Madrid, Spain; 3 Laboratorio de Enfermedades Mitocondriales y Neuromusculares, Instituto de Investigación Sanitaria Hospital 12 de Octubre (imas12), 28041 Madrid, Spain; 4 Centro de Investigación Biomédica en Red de Enfermedades Raras (CIBERER), 28029 Madrid, Spain; 5 Department of Genetics & Genomics, Instituto de Investigación Sanitaria-Fundación Jiménez Díaz University Hospital, Universidad Autónoma de Madrid (IIS-FJD, UAM), 28040 Madrid, Spain

**Keywords:** iPSCs, autosomal dominant optic atrophy, ADOA, *OPA1*, retinal ganglion cells, RGCs, CRISPR/Cas9, disease modeling, isogenic control

## Abstract

Autosomal dominant optic atrophy (ADOA) is a rare progressive disease mainly caused by mutations in *OPA1*, a nuclear gene encoding for a mitochondrial protein that plays an essential role in mitochondrial dynamics, cell survival, oxidative phosphorylation, and mtDNA maintenance. ADOA is characterized by the degeneration of retinal ganglion cells (RGCs). This causes visual loss, which can lead to legal blindness in many cases. Nowadays, there is no effective treatment for ADOA. In this article, we have established an isogenic human RGC model for ADOA using iPSC technology and the genome editing tool CRISPR/Cas9 from a previously generated iPSC line of an ADOA plus patient harboring the pathogenic variant NM_015560.3: c.1861C>T (p.Gln621Ter) in heterozygosis in *OPA1*. To this end, a protocol based on supplementing the iPSC culture media with several small molecules and defined factors trying to mimic embryonic development has been employed. Subsequently, the created model was validated, confirming the presence of a defect of intergenomic communication, impaired mitochondrial respiration, and an increase in apoptosis and ROS generation. Finally, we propose the analysis of *OPA1* expression by qPCR as an easy read-out method to carry out future drug screening studies using the created RGC model. In summary, this model provides a useful platform for further investigation of the underlying pathophysiological mechanisms of ADOA plus and for testing compounds with potential pharmacological action.

## 1. Introduction

Autosomal dominant optic atrophy (known as ADOA, OMIM #165500) is a rare progressive disease and the most common form of hereditary optic neuropathy, with an estimated prevalence of 3/100,000 in the general population [1]. ADOA is mainly caused by pathogenic variants in *OPA1*, a nuclear gene that encodes for a multifunctional protein located in the mitochondrial inner membrane. OPA1 plays an essential role in maintaining the shape and structure of the mitochondria, mitochondrial DNA maintenance, cell survival, and oxidative phosphorylation [2,3]. To date, more than 400 pathogenic variants in the *OPA1* gene have been described as associated with DOA [4,5], leading to decreased energy production capacity and the degeneration of retinal ganglion cells (RGCs), which produces optic nerve atrophy [6]. Therefore, patients present with optic atrophy characterized by the presence of central scotomas, optic disc pallor, and dyschromatopsia. This causes a progressive visual loss that can lead to legal blindness [7,8]. Moreover, approximately 20% of ADOA patients show additional extra ophthalmological symptoms such as hearing loss, neuropathy, myopathy, or ataxia, among others; this form of the disease is known as ADOA plus [9].

Nowadays, there is no effective treatment for this disease. Several compounds, including idebenone, have been tested with the objective of diminishing the oxidative damage. Although a recent study showed that idebenone administration in DOA patients could improve visual function, the efficacy of the compound turns out to be low when it causes damage to a huge number of RGCs [10]. Therefore, additional strategies need to be explored to find an effective treatment for these patients.

In this sense, the discovery of induced pluripotent stem cells (iPSCs) in 2006 by Dr. Yamanaka was a major breakthrough in biomedical research [11]. iPSCs can be generated in vitro by reprogramming somatic cells thanks to the ectopic expression of four transcription factors: OCT3/4, SOX2, KLF4 and C-MYC. These cells have morphological features similar to those of embryonic stem cells (ESCs), since they are able to self-renew indefinitely in culture and are also pluripotent, so they have the potential to differentiate towards any cell type of the three germ layers [12,13]. The possibility of generating iPSC lines establishes them as a platform with huge potential for different applications, highlighting their use as a source to obtain cell products for cell therapy approaches [14] or the likelihood of generating disease models to test the efficacy of specific compounds [15]. For disease modeling, it is necessary to differentiate patient-derived iPSC lines towards the affected cell type in the disease. Over the years, several different protocols have been established to generate RGCs, starting with iPSCs. Some of them consist of the overexpression of some key transcription factors for RGC development (such as Atoh7). These protocols are faster and more efficient, but they have the associated risk of integration into the genome [16]. Conversely, the majority of the methods are based on supplementing the culture medium with small molecules to mimic embryonic development. Typically, these protocols are longer and give rise to a heterogeneous retinal cell population, but the use of viral vectors is not required, which could make possible its future transfer to the clinic [17].

Another key aspect of the generation of in vitro disease models is the selection of the most appropriate control. In this regard, the CRISPR/Cas9 genome editing tool has marked a milestone due to its effectiveness, relatively low cost, and simplicity [18]. Among its huge number of applications, the CRISPR/Cas9 system enables the specific editing of the desired nucleotides for the correction of the pathogenic variants to establish an ideal isogenic control for the model [19].

In this article, a human isogenic RGC model for ADOA plus based on iPSC technology has been generated. For this purpose, the previously generated Oex2054SV.4 iPSC line (Oex20.4) harboring the pathogenic variant NM_015560.3: c.1861C>T (p.Gln621Ter) in the *OPA1* gene [20] was corrected with the CRISPR/Cas9 system to generate an isogenic control. Then, the patient iPSC line and its matched control were successfully differentiated towards RGCs for the creation of a human personalized in vitro model. This model has been characterized and validated, providing new insights about the etiopathogenesis of the disease. Finally, we propose an *OPA1* expression rescue assay as an easy read-out drug screening method that can be employed with the created isogenic RGC model for the search for a personalized treatment for this disabling disease.

## 2. Results

### 2.1. Gene Editing with CRISPR/Cas9

In order to generate an isogenic control, the pathogenic variant NM_015560.3: c.1861C>T (p.Gln621Ter) in the *OPA1* gene was corrected in the iPSC line Oex20.4 with the CRISPR/Cas9 technology. For this purpose, two different single guide RNAs (sgRNAs) were designed as described in the Section 4.2.1, both containing the mutation in their sequence to enhance the editing efficiency. Figure S1 shows the location of both sgRNAs and their PAM sequences. Then, to evaluate the cutting efficiency of both guides, an endonuclease T7E1 assay was performed. The cutting efficiency was calculated as described in Section 4.2.4. and was 12% for sgRNA.1 and 7% for sgRNA.2 (Figure S2), so sgRNA.1 was selected as the most appropriate guide to continue with the editing strategy.

Hereafter, a single-stranded oligodeoxynucleotide (ssODN) was designed as a repair template, since it has been previously demonstrated that a ssODN in combination with ribonucleoprotein complexes (RNPs) is a highly effective way to introduce single nucleotide changes via homology-directed repair (HDR). To assess editing efficiency, a Restriction Fragment Length Polymorphism (RFLP) analysis was performed by introducing a cut site for the restriction enzyme XmnI after gene editing with the designed oligodeoxynucleotide. Thus, without edition, XmnI cuts the PCR amplicon just in one of the alleles, given that it is a heterozygous pathogenic variant. Thus, fragments of 641 bp, 555 bp, and 86 bp will be observed. On the contrary, if gene editing has taken place correctly, XmnI generates two fragments of 86 bp and 555 bp. After the analysis, the cutting percentage in the pool without editing was 30%, while it was 45% in the edited cells. Thereby, the editing efficiency was 15%, approximately (Figure S3).

**Figure 1:**
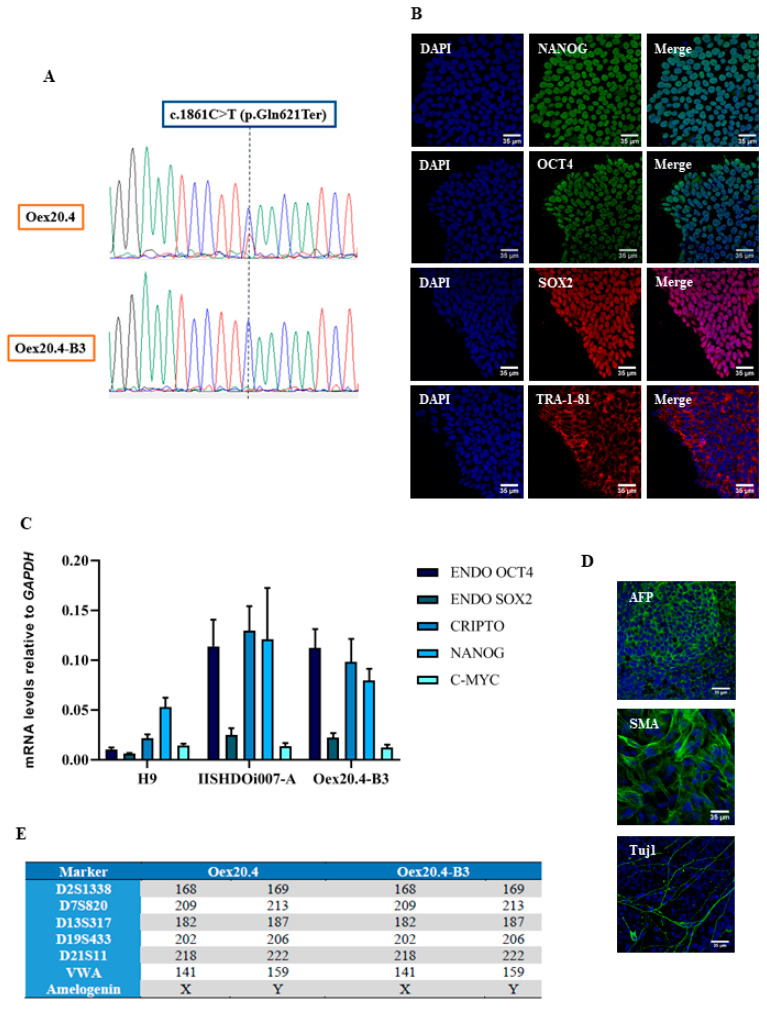
Pluripotency and integrity assessment of the iPSC line Oex20.4-B3. (**A**) Gene editing confirmation of Oex20.4-B3 by Sanger sequencing. The iPSC line Oex20.4 harboring the pathogenic variant c.1861C>T in *OPA1* is shown above, while the edited line Oex20.4-B3 is below it. (**B**) Immunocytochemistry assay showing the positive expression of the pluripotency markers SSEA4, SOX2, OCT4, TRA-1-81, and TRA-1-60. Scale bars: 35 µm. (**C**) RT-qPCR analysis showing the positive expression of the markers *OCT4*, *SOX2*, *CRIPTO*, *NANOG*, and *C-MYC* compared to the human embryonic stem cell line H9 and to the previously published iPSC line IISHDOi007-A [22]. Values represent the mean of at least three independent replicates and were normalized to the *GAPDH* gene. Error bars represent the standard deviation. (**D**) Immunocytochemistry analysis of lineage-specific markers showing positive expression for AFP (endoderm), SMA (mesoderm), and Tuj1 (ectoderm). Scale bars: 35 µm. (**E**) DNA fingerprinting analysis demonstrating the genetic identity of the edited iPSCs Oex20.4-B3 and the iPSC line Oex20.4. (**E**).

Then, subcloning of potentially edited iPSCs was performed, and, after analyzing the clones by RFLP for the verification of gene editing, 10 clones were found to be positive. One of the 10 clones, named Oex20.4-B3, was selected for further experiments based on morphological and growth criteria and was expanded. Subsequently, a confirmation of the correction of the pathogenic variant was performed by Sanger sequencing (Figure 1A). One of the main disadvantages of the CRISPR/Cas9 system is the possibility of modifying the sequence of undesired targets. We tried to minimize this with several strategies, such as delivering the CRISPR system using RNPs, using a high-fidelity Cas9, or carefully designing the sgRNA. In spite of this, we carried out an off-target analysis by using prediction algorithms, and the employed sgRNA had only one possible off-target with four missmatches, which drastically diminishes the Cas9 cutting efficiency [21]. Moreover, it was located in a non-coding region of the genome.

Finally, to evaluate if the corrected iPSC line Oex20.4-B3 has preserved its pluripotency and integrity after the editing process, a complete battery of tests was carried out. First, the expression of the pluripotency markers NANOG, OCT4, SOX2, and TRA-1-81 was confirmed by immunocytochemistry (Figure 1B). In addition, a quantitative RT-qPCR analysis also showed the pluripotency of the line by the positive expression of the stemness markers *OCT4*, *SOX2*, *CRIPTO*, *NANOG*, and C-*MYC*. Total RNA from human embryonic stem cells (H9) (Celprogen, Torrance, CA, USA; #36101) and the iPSC line IISHDOi007-A [22] were used as a reference for the gene expression levels of stemness markers (Figure 1C). Then, an in vitro differentiation assay was employed in order to check the potential of the iPSCs to give rise to cells of the three germ layers. As shown in Figure 1D, the iPSC line Oex20.4-B3 correctly differentiated towards endoderm (positive for AFP), mesoderm (positive for SMA), and ectoderm (positive for Tuj1). Moreover, a DNA fingerprinting analysis was performed to prove the genetic identity of the edited iPSC lines Oex20.4-B3 and Oex20.4 iPSCs (Figure 1E). Finally, the line Oex20.4-B3 was checked to be mycoplasma-free and showed a normal karyotype 46, XY (Figure S4) discarding the presence of karyotypic abnormalities generated after the edition process.

### 2.2. iPSC Differentiation towards RGCs

**Figure 2:**
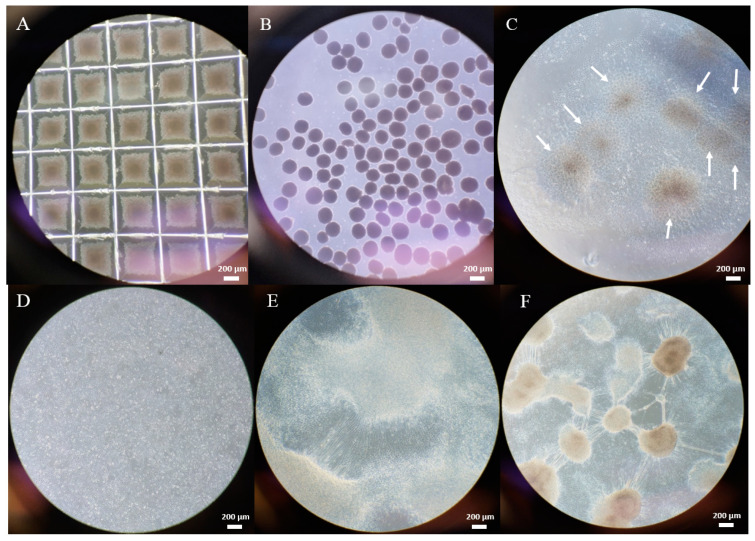
Differentiation process of iPSCs towards RGCs. Bright field representative images show the main cellular structures generated during the process. (**A**) Oex20.4-B3 forming EBs on the second day of differentiation in the Aggrewell^TM^ 800 plate (4×). The EBs start acquiring a rounded shape. (**B**) Oex20.4-B3 EBs at day 4 of differentiation with defined borders. (**C**) Neural rosettes (pointed with white arrows) of Oex20.4-B3 at day 8 of differentiation (4×). (**D**) Oex20.4 RPCs isolated from the neural rosettes (4×). (**E**) Oex20.4 cells with typical morphology of RGCs extending their axons that start emerging at day 18 of the differentiation process from RPCs. (**F**) Oex20.4 RGCs at day 21 with the characteristic generation of interconnected cell groups by neuronal processes at the final stage of the process. Scale bars: 200 µm.

For the generation of an in vitro model of DOA disease, the iPSC line Oex20.4 and its matched isogenic control were differentiated towards RGCs. To do that, the protocol previously described by Lee et al. (2018) [23] with minor modifications has been employed, as described in the Section 4. Firstly, embryoid bodies (EBs) were formed while we treated them with the required activators and inhibitors of different signaling pathways for neuroectoderm induction. We treated them with dorsomorphin, a BMP pathway inhibitor, and SB431542, a TGF-β pathway inhibitor [24]. Moreover, the addition of the Wnt pathway inhibitor XAV939 plays an essential role in the determination of neural retinal identity [25], along with the culture supplementation with IGF-I [26]. In Figure 2A, the formation of the EBs is shown, which are acquiring its rounded shape. At day 4th, EBs are completely formed (Figure 2B) and are cultured in adherent conditions and treated with a high dose of FGF-2, which promotes the induction of eye-field-specific TFs. As shown in Figure 2C, neural rosette structures are generated, which are highly dense multicellular structures that are very organized, presumptively containing retinal progenitor cells (RPCs). On day 8, those neural rosettes were ready to be isolated to enrich the retinal progenitor cells (RPCs). At this point, RPCs could be efficiently isolated and could be expanded and cryopreserved (Figure 2D) [23].

Finally, RPCs were differentiated from RGCs. For this purpose, we employed DAPT, a Notch pathway inhibitor, since this pathway was repressed in retinal development prior to RGC generation [27]. After DAPT addition, cells start acquiring a neuronal-like aspect with a triangular soma and expanding long projections and dendrites (Figure 2E). For RGC maturation, the culture is supplemented with the brain-derived neurotrophic factor (BDNF) for 3–4 weeks, which enhances neural survival [28]. In Figure 2F, it is shown that, at the end of the process, the generated RGCs establish several connections through neuronal processes, being these structures typical of the last stages of differentiation to RGCs [23].

**Figure 3:**
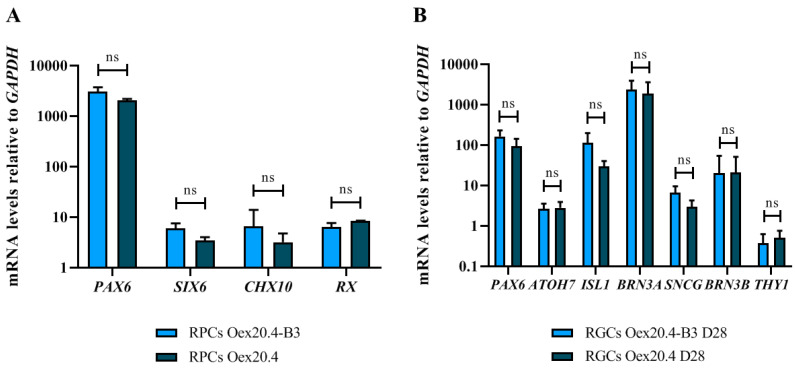
Quantitative PCR analysis to evaluate the relative expression of typical genes in iPSC-derived RPCs and RGCs. (**A**) We analyzed the expression of different ocular lineage genes such as *PAX6*, *SIX6*, *CHX10*, and *RX*. (**B**) We analyzed the expression of typical RGC genes such as *ATOH7*, *BRN3A*, *BRN3B*, *SNCG*, *ISLET1*, and *THY1* on day 28 of differentiation. The values represent the mean of at least three replicates, and they are relative to the expression of the housekeeping gene GAPDH and to the expression values of the starting iPSCs for each gene. Error bars show the standard deviation. ns: no significant differences (Mann-Whitney U test statistical analysis).

In order to evaluate if the differentiation process had taken place successfully, we analyzed by RT-qPCR the expression of several genes in distinct temporal stages of the differentiation process towards RGCs. Firstly, once RPCs were isolated and expanded from the neural rosette structures, they were analyzed to detect the expression of typical markers of this intermediate cell stage. As shown in Figure 3A, the RPCs generated from Oex20.4 and Oex20.4-B3 iPSCs were positive for the typical ocular lineage markers PAX6, SIX6, CHX10, and RX. Moreover, expression values were similar for both lines, without significant differences between them. Then, we analyzed the expression values of typical RGC genes by RT-qPCR at the end of the differentiation towards RGCs. Oex20.4 and Oex20.4-B3 RGCs showed expression of *ATOH7*, *BRN3A*, *BRN3B*, *SNCG*, *ISLET1*, and *THY1* (Figure 3B). Remarkably, statistically significant differences in the expression values of RGCs between both lines were not observed, which suggests that the pathogenic variant c.1861C>T (p.Gln621Ter) in *OPA1* does not affect the differentiation process towards RGCs.

**Figure 4:**
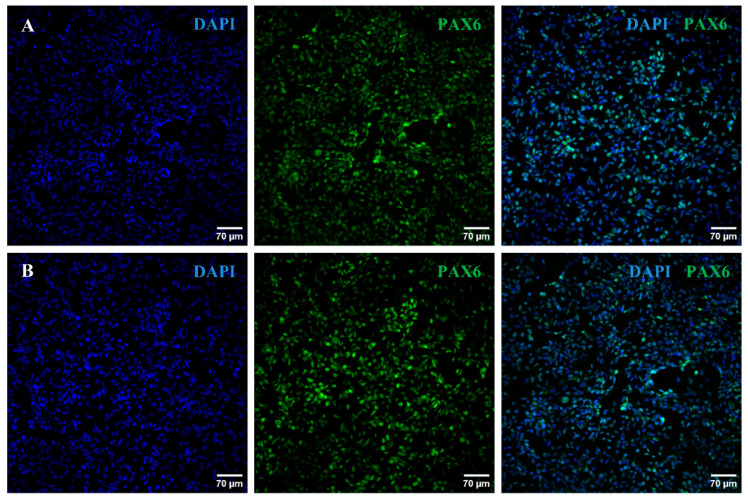
Immunocytochemistry analysis of Oex20.4 and Oex20.4-B3 RPCs. Confocal images showing the positive staining of PAX6 (green) for Oex20.4 (**A**) and Oex20.4-B3 (**B**) after isolating neural rosettes and RPC expansion. Nuclei were counterstained with DAPI (blue). Scale bars: 70 µm.

**Figure 5:**
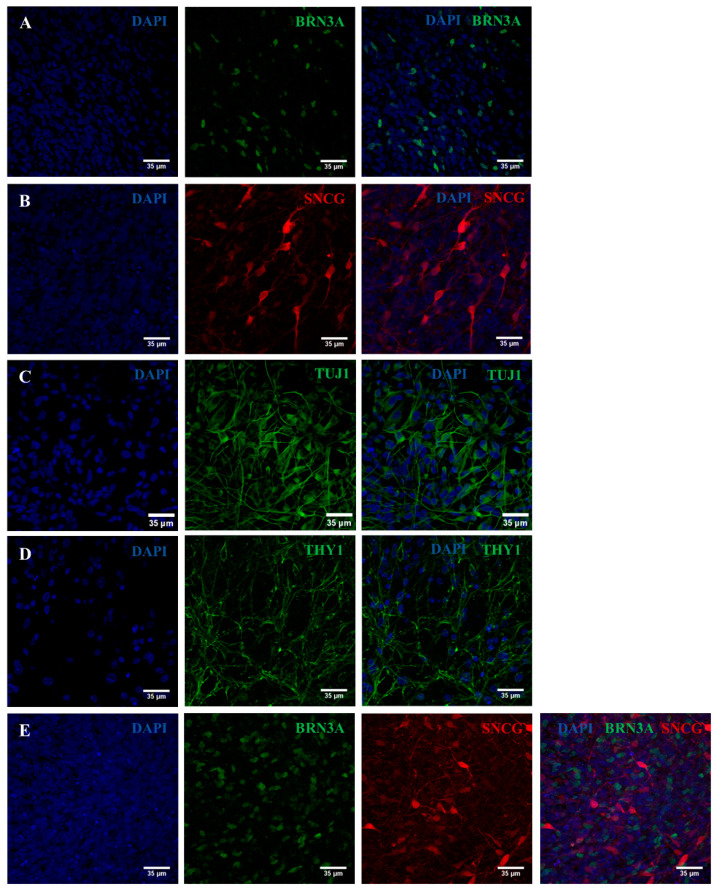
Immunocytochemistry analysis to evaluate the expression of typical RGC markers. Representative confocal images show the positive expression of RGC markers for the line Oex20.4-B3 at the end of the differentiation process. (**A**) Positive expression for BRN3A (green). (**B**) SNCG (red). (**C**) TUJ1 (green). (**D**) THY1 (green). (**E**) BRN3A (green) and SNCG (red). Nuclei were counterstained with DAPI (blue). Scale bars: 35 µm.

**Figure 6:**
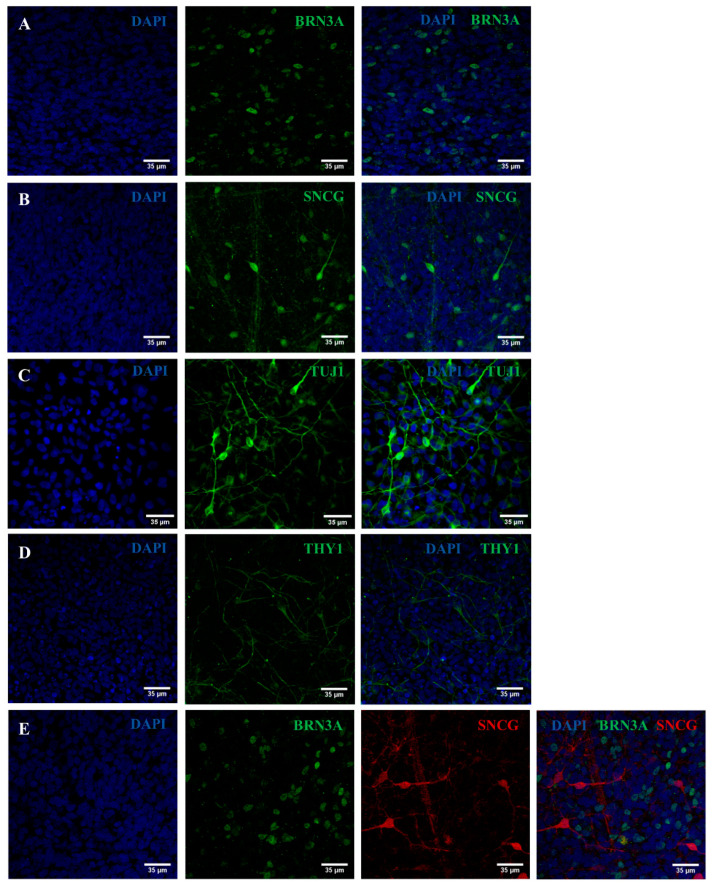
Immunocytochemistry analysis to evaluate the expression of typical RGC markers. Representative confocal images show the positive expression of different RGC markers for the line Oex20.4 at the end of the differentiation process. (**A**) Positive expression for BRN3A (green). (**B**) SNCG (red). (**C**) TUJ1 (green). (**D**) THY1 (green). (**E**) BRN3A (green) and SNCG (red). Nuclei were counterstained with DAPI (blue). Scale bars: 35 µm.

In parallel, we assessed by immunocytochemistry the expression of PAX6, a typical intermediate marker of RPCs. As shown in Figure 4, Oex20.4 and Oex20.4-B3 RPCs were positively stained for PAX6. Then, we also assessed the expression of some typical RGC markers at day 28 of the process by an immunocytochemistry analysis. RGCs derived from Oex20.4 and the isogenic control were positive for the markers BRN3A, ɣ-synuclein (SNCG), TUJ1, and THY1 (Figure 5 and Figure 6).

### 2.3. Validation of the Created RGC-Based Model

To delve into the etiopathogenesis of DOA and validate the generated RGC model, several assays were performed: (1) analysis of OPA1 expression at the mRNA and protein levels; (2) analysis of the presence of depletion or multiple deletions in the mtDNA; (3) oxygen consumption analysis; (4) measurement of apoptosis; and (5) ROS detection.

#### 2.3.1. Analysis of OPA1 Expression

**Figure 7:**
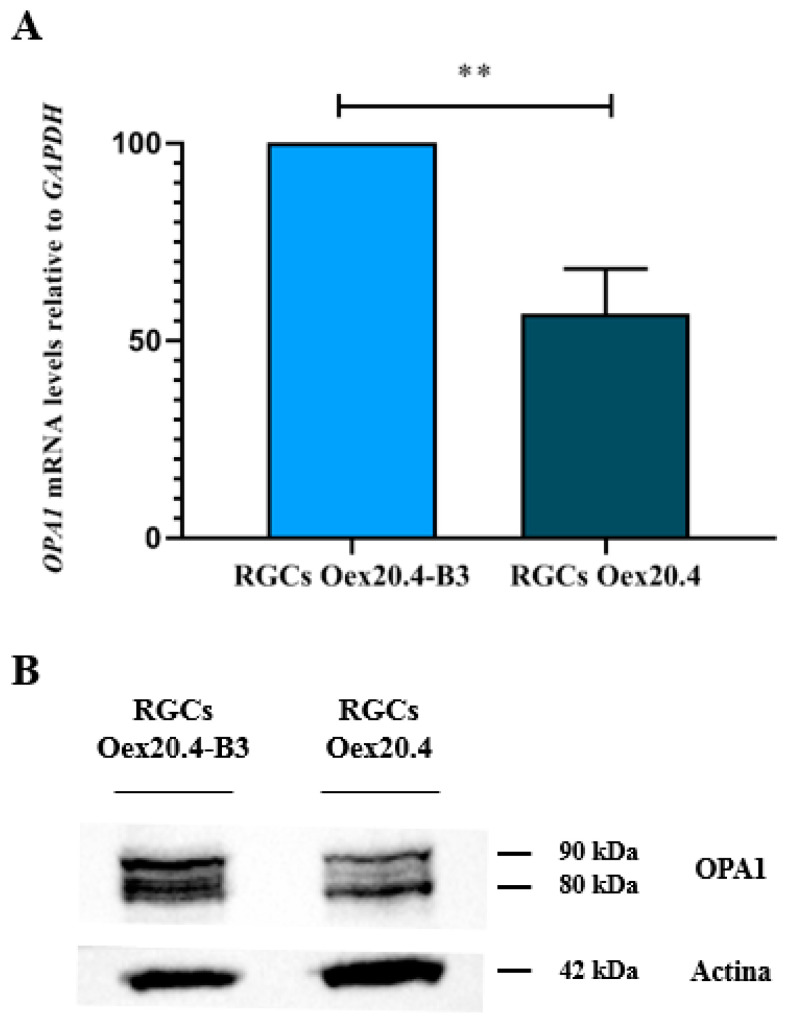
Analyses of OPA1. (**A**) Quantitative PCR analysis for the evaluation of *OPA1* relative expression in the generated RGCs. Oex20.4 RGCs showed a significantly diminished expression in comparison to the control RGCs. The values are represented as percentages. The value of the control line is set to 100% of its expression. The values represent the mean of at least three replicates, and they are relative to the expression of the housekeeping gene *GAPDH*. Error bars show the standard deviation. ** *p*-value < 0.01 (Student’s *t* test statistical analysis). (**B**) Western blot for OPA1 in the RGCs. Oex20.4 RGCs showed approximately a 50% reduction in OPA1 quantity (90 kDa isoform) with regard to Oex20.4-B3 RGCs. Actin was employed as a loading control (42 kDa).

Firstly, *OPA1* expression was quantified by RT-qPCR in Oex20.4 and Oex20.4-B3 RGCs, using *GAPDH* as a housekeeping gene. Figure 7 shows that, at the end of differentiation, *OPA1* expression is decreased by approximately 50% in the patient RGCs in comparison with the isogenic control RGCs. A Western blot assay also confirmed the presence of OPA1 protein in Oex20.4-B3 RGCs, and those protein levels are reduced in the RGCs derived from the patient. After quantifying the isoform of interest (90 kDa), the values of relative intensities were 0.84 for Oex20.4-B3 and 0.4 for Oex20.4. Therefore, OPA1 protein quantity is reduced by approximately 50% in the patient RGCs versus the control one.

#### 2.3.2. Analyses of mtDNA Multiple Deletions and Depletion

**Figure 8:**
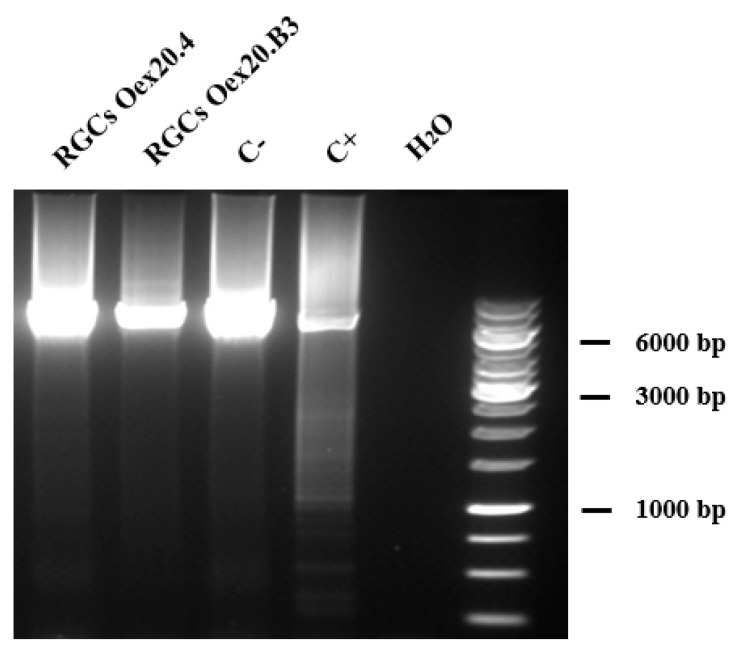
Long-range PCR for the evaluation of multiple deletions in the mtDNA. Oex20.4 and Oex20.4-B3 RGCs do not harbor deletions in the mtDNA. RGCs Oex20.4: patient-derived RGCs; RGCs Oex20.4-B3: isogenic control-derived RGCs; C−: negative control; C+: positive control (mtDNA from a patient presenting with multiple deletions).

In order to evaluate if the pathogenic variant c.1861C>T (p.Gln621Ter) in *OPA1* affects the mtDNA maintenance, the presence of multiple deletions and depletion in the mtDNA of Oex20.4 and Oex20.4-B3 RGCs has been analyzed. For this purpose, a fragment of the mitochondrial genome of Oex20.4 and Oex20.4-B3 RGCs, where deletions are commonly located in the mtDNA genome, was amplified by long-range PCR, and the product was separated by electrophoresis to analyze the presence of mtDNA deletions. As shown in Figure 8, multiple deletions in the mtDNA were not detected in the patient RGCs.

**Figure 9:**
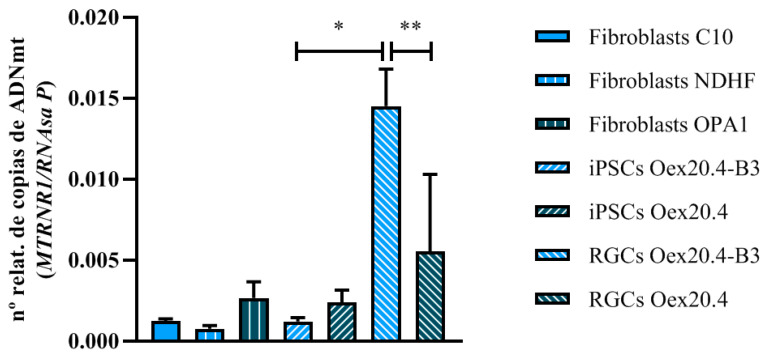
mtDNA depletion analysis with qPCR. Oex20.4 RGCs showed a diminished relative mtDNA copy number in comparison with isogenic control RGCs. The values represent the mean of at least three replicates. Error bars show the standard deviation. ** *p*-value < 0.01; * *p*-value < 0.05 (Mann-Whitney U test statistical analysis).

Moreover, the relative content of mtDNA was determined by real-time qPCR to evaluate the presence of depletion in the mtDNA, for which the mitochondrial gene *MT-RNR1* was analyzed and the values were normalized against the single-copy nuclear gene *RNase P*. Overall, mtDNA depletion in a tissue does not imply depletion in another tissue. In fact, mtDNA depletion is frequently restricted to specific tissues. In our DOA patient, RGCs are the main affected target cell type, and, for that reason, it could be more probable to detect mtDNA depletion in RGCs than in other tissues. To demonstrate this point, we have analyzed cell types other than RGCs, such as fibroblasts of the patient that rely more on OXPHOS and iPSCs with a low dependence on OXPHOS. As shown in Figure 9, the relative mtDNA copy number in Oex20.4 RGCs was significantly diminished in comparison with the mtDNA copy number in the control RGCs, indicating a depletion of the mtDNA in the patient RGCs. Furthermore, there was a significant increase in copy number in Oex20.4-B3 RGCs compared with the starting iPSCs, whereas, when comparing iPSCs and RGCs from the mutant line, the differences were not statistically significant. With these results, we demonstrate the suitability of the created RGC model, which also presents a typical defect of intergenomic communication.

#### 2.3.3. Analysis of the Mitochondrial Respiration

**Figure 10:**
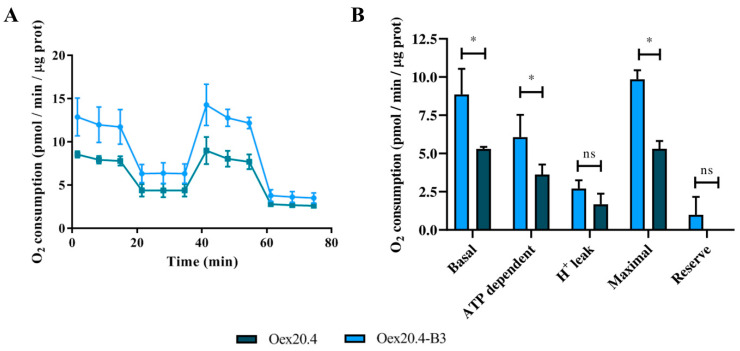
Bioenergetic analysis of the generated RGCs using a Seahorse analyzer. (**A**) Graph showing mitochondrial respiration reflected by OCR levels. (**B**) Calculated rates of basal respiration, ATP-linked respiration, proton leak, maximal respiratory capacity and reserve respiration. Oex20.4 RGCs showed significantly reduced basal, ATP dependent and maximal respiration when compared with Oex20.4-B3 RGCs. Values of O_2_ consumption (pmol/min) normalized to protein quantity (µg) are shown. The values represent the mean of at least three independent replicates, and error bars show the standard deviation. * *p*-value < 0.05; ns: no significant differences (Mann-Whitney U test statistical analysis).

With the aim of evaluating if the pathogenic variant has some effect on the bioenergetic function of RGCs, we tested the mitochondrial respiration using a Seahorse analyzer. As shown in Figure 10, we could observe that some of the parameters, including basal, ATP-dependent, and maximal respiration, were significantly reduced in the patient-derived RGCs compared to the isogenic control one. This indicates that the pathogenic variant produces an impairment of the bioenergetic function in the patient RGCs.

#### 2.3.4. Quantification of Reactive Oxygen Species (ROS) Generation

**Figure 11:**
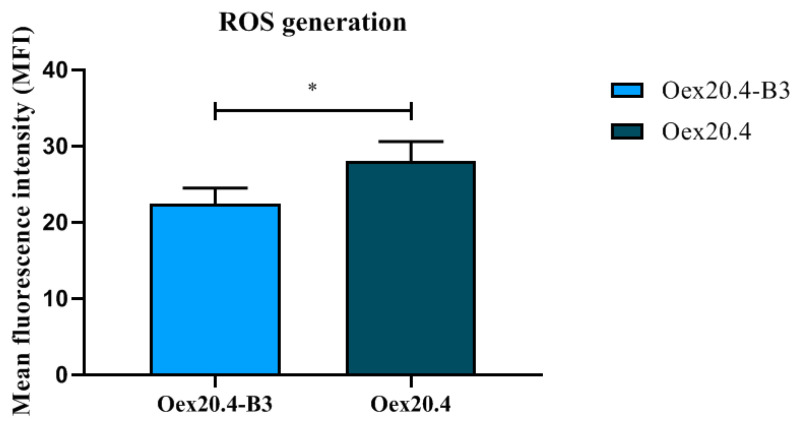
Quantification of ROS generation by flow cytometry. Oex20.4 RGCs showed a significantly higher percentage of MFI in comparison with Oex20.4-B3 RGCs. The values represent the mean of at least three independent replicates, and error bars show the standard deviation. * *p*-value < 0.05 (Student *t* test statistical analysis).

We used the probe 2′-7′-dihydrochlorofluorescein diacetate (DCFH-DA) to evaluate the generation of reactive oxygen species (ROS). RGCs at the end of the differentiation process were stained with DCFH-DA 1 µM for 10 min, and the signal was detected by flow cytometry. The RGCs from the DOA patient showed a significantly higher mean fluorescence intensity (MFI) than the RGCs from the isogenic control (Figure 11), which is indicative of increased oxidative damage in these cells.

#### 2.3.5. Apoptosis Quantification

**Figure 12:**
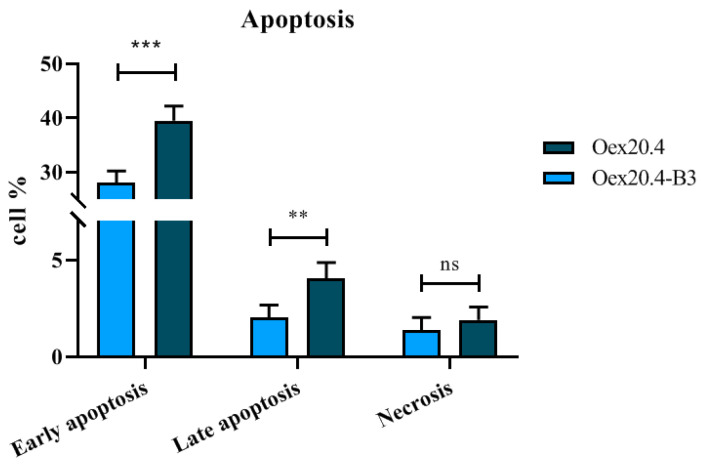
Apoptosis quantification by flow cytometry. Oex20.4 RGCs showed significantly higher early and late apoptosis than Oex20.4-B3 RGCs. The values represent the mean of at least three independent replicates, and error bars show the standard deviation. *** *p*-value < 0.001; ** *p*-value < 0.01; ns: no significant differences (Student *t* test statistical analysis).

For apoptosis assessment, double staining with Annexin V and propidium iodide (PI) was used in Oex20.4 and Oex20.4-B3 RGCs at the end of the differentiation process and detected by flow cytometry. As shown in Figure 12, the patient RGCs presented both early and late apoptosis that was significantly augmented in comparison with the control RGCs. For necrosis, no differences were observed. Therefore, patient-derived RGCs show an increased susceptibility to programmed cell death.

### 2.4. Nicotinamide Potential for the Rescue of OPA1 Expression

The generated human RGC model of ADOA plus harboring the pathogenic variant c.1861C>T (p.Gln621Ter) in OPA1 recapitulates the main biochemical features of the disease, and therefore it could be employed as a future platform to perform high-throughput drug screenings. For this purpose, the availability of an easy read-out screening method would be very valuable.

**Figure 13:**
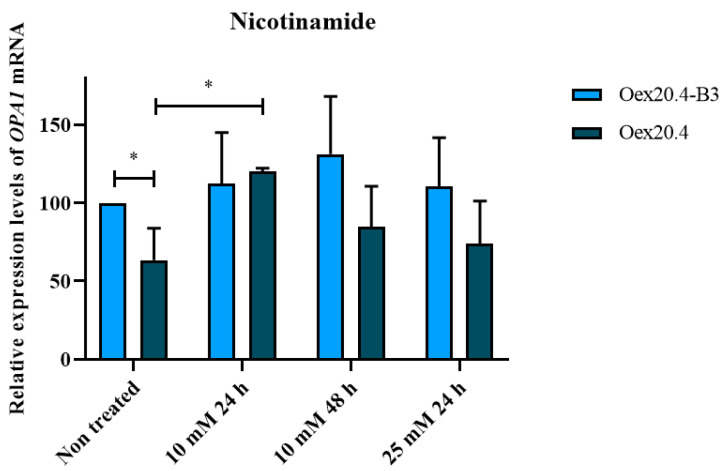
RT-qPCR analysis of *OPA1* expression rescue with nicotinamide. Oex20.4 and Oex20.4-B3. RGCs were treated with 10 mM nicotinamide for 24 and 48 h and 25 mM for 24 h. The treatment with 10 mM of nicotinamide for 24 h significantly increased *OPA1* expression in Oex20.4 RGCs. Expression is shown in percentage, considering 100% of expression for the control, non-treated line. The values represent the mean of at least three independent replicates and are relative to *GAPDH* gene expression. Error bars show the standard deviation. * *p*-value < 0.1 (Student *t* test statistical analysis).

In this study, the potential of nicotinamide or vitamin B3 to rescue OPA1 expression in the patient’s RGCs has been evaluated. To this purpose, Oex20.4 and Oex20.4-B3 iPSCs have been differentiated towards RGCs and treated with 10 mM nicotinamide for 24 and 48 h and 25 mM for 24 h, respectively. We observed by RT-qPCR that the treatment with 10 mM nicotinamide for 24 h increased *OPA1* expression in Oex20.4 RGCs, bringing the value closer to the control RGCs (Figure 13). This result shows that the analysis of *OPA1* expression rescue by RT-qPCR could be very useful to be employed as a simple read-out method to perform drug screenings to search for an effective treatment for ADOA.

## 3. Discussion

Autosomal dominant optic atrophy (ADOA) is a mitochondrial disease mainly caused by the degeneration of retinal ganglion cells (RGCs), which can provoke legal blindness in many cases [1,6]. Approximately 20% of the patients present with a syndromic form of the disease known as ADOA plus [9]. Until date, there has been no effective treatment for ADOA. In this sense, iPSC technology makes it possible to generate patient-specific disease models since these cells can be differentiated towards the affected cell type, which could be a valuable tool for the search for a therapy against this disease [15]. In this article, we present the establishment of an isogenic human iPSC-based RGC model for ADOA plus. To this aim, a previously generated ADOA plus iPSC line harboring the pathogenic variant c.1861C>T (p.Gln621Ter) in the *OPA1* gene [20] and its matched isogenic control, created with the CRISPR/Cas9 system to eliminate the genetic background, have been used.

Afterwards, both the ADOA plus iPSC line and its matched isogenic control were differentiated towards RGCs, the mainly affected target cell type in the disease. Most of the differentiation protocols that have been described to date consist of 2D cultures where cells are specifically directed towards the RGC lineage. In recent years, several groups have focused on the generation of the so-called retinal “organoids”, 3D culture systems that recapitulate the process of retinal genesis more closely than a monolayer culture [29]. However, despite the many applications of retinal organoids, their usefulness in modeling ADOA is limited because they contain numerous cell types that are not affected by the disease and a low number of RGCs [30]. For this reason, we decided to employ a 2D RGC differentiation protocol. The existing 2D differentiation protocols are mainly divided into two groups. Some methods are based on the genetic modification of iPSCs through the overexpression of certain transcription factors (TFs), such as Atoh7 [16]. These strategies typically achieve high efficiency, enabling them to obtain RGCs quickly. Nevertheless, the main drawback is the possible integration of the employed viral vectors into the genome. On the other side, most protocols are based on the addition of small molecules to the culture media to modulate certain signaling pathways, imitating what happens in embryonic development. Although these approaches turn out to be generally slower and have lower efficiencies, they are also safer as they do not require the use of vectors, which will facilitate future translation to the clinic [17]. For that reason, the protocol described by Lee et al. in 2018 [23] with minor modifications has been selected for the creation of the present model. Using this methodology, firstly, RPCs obtained from Oex20.4 and its isogenic control with positive expression of the typical ocular markers PAX6, CHX10, SIX6, and RX have been obtained. After the second part of the differentiation process, we could differentiate those RPCs towards RGCs, which showed positive expression for the typical markers ATOH7, BRN3A, BRN3B, SNCG, ISLET1, β-III-tubulin, and THY1. ATOH7 exhibits low expression since its function is to regulate the determination towards RGCs by activating genes such as BRN3A, BRN3B, or ISLET1. Thus, probably in this final stage, its expression does not show its higher value. Regarding THY1, the expression values are lower than the other typical RGC genes. The reason could be that, even though THY1 is a marker of mature RGCs, it is also expressed in other cell types, including stem cells [31]. In fact, when comparing RGCs and iPSC expression, the THY1 value appears close to the unit.

Remarkably, we did not observe differences in the marker expression levels between patient and control-derived RGCs therefore, the variant does not affect the differentiation process. This is in line with the observations made by Sladen and colleagues in 2022 in their generated ADOA models. They successfully differentiated several iPSC lines carrying *OPA1* variants towards RGCs: a heterozygous knockout (OPA1^+/−^), the heterozygous deletion c.2708_2711delTTAG (p.Arg905Ter) from a patient with ADOA, and the missense variant c.1334G>A (p.Arg445His) from an ADOA plus patient [32]. However, Chen et al. (2016) differentiated an iPSC line harboring the intronic variant c.2496+1G>T, and they observed a diminished differentiation efficiency [33]. These data suggest that each pathogenic variant in the *OPA1* gene could behave in a different way.

Then, the generated model was characterized and validated from a functional point of view in order to check if it mimicked the main biochemical aspects of ADOA. OPA1 plays an essential role in mtDNA maintenance; thus, mutations in this gene can produce a deregulation of its integrity and quantity [34]. Numerous cases of different mutations in *OPA1* have been described, resulting in multiple deletions or mtDNA depletion. For instance, it was observed that the missense variant c.1635C>G (p.Ser545Arg) in the GTPase domain of OPA1 triggers multiple deletions [35], while the missense variant c.1187T>G (p.Leu396Arg) in the same domain produces mtDNA depletion [36]. Regarding pathogenic variants in other domains, the nonsense mutation c.2729T>A (p.Val910Asp), located in the effector final domain, gives rise to multiple deletions [37]. Another example of nonsense mutation, the variant c.112C>T (p.Arg38Ter), reported by Kim et al. at the beginning of the GTPase domain, results in mtDNA depletion [36]. In the case of the variant under study, we compared the relative mtDNA copy number between the patient RGCs and its matched isogenic control, since RGCs are the affected target cell type in ADOA. This way, we confirmed the presence of a defect in intergenomic communication, as Oex20.4 RGCs showed depletion of the mtDNA. Regarding the mtDNA copy number present in the different cell types analyzed, a significant increase in copies could be observed in RGCs from Oex20.4-B3 compared to the starting iPSCs, which did not occur in the case of the Oex20.4 line. This observation is consistent with the fact that RGCs present a more oxidative metabolism than iPSCs, which are more mitotically active, proliferating cells. Furthermore, iPSCs are known to be highly glycolytic mainly because they present an immature mitochondrial network with underdeveloped cristae, indicative of lower mitochondrial activity [38,39]. Even so, in the case of the mutant line, the copy number differences were not significant between the RGCs and the starting iPSCs, due to the presence of the intergenomic communication defect.

Moreover, it has been previously demonstrated that mutations in *OPA1* cause high levels of reactive oxygen species (ROS) and oxidative damage [40], as well as an increase in apoptosis susceptibility since OPA1 plays a role in cytochrome c release [3]. In line with that, a significant increase in ROS generation and in the percentage of apoptotic cells was observed in patient RGCs compared to control RGCs, with no difference in the percentage of necrotic cells. For both lines, the percentage of cells in early apoptosis is significantly higher than the percentage of cells in late apoptosis, and this slightly higher than the percentage of necrotic cells. This is indicative of the sequential process that cell death follows: first, when cell homeostasis is disrupted, apoptosis, or programmed cell death, occurs, characterized by the translocation of phosphatidylserine to the plasma membrane for the recognition of damaged cells by phagocytes. This is followed by condensation and fragmentation of the nucleus and cytoplasm and the formation of apoptotic bodies in later apoptosis. When cell damage is very high and there is a loss of control of cell functions, necrosis occurs, a more disorganized death characterized by membrane rupture and subsequent release of plasma contents, triggering an inflammatory response [41]. Therefore, the susceptibility to apoptosis experienced by RGCs in ADOA could eventually lead to necrosis, although there is little evidence that this type of cell death occurs and further studies are needed [42,43]. One possibility is that, in culture, we can only observe the difference in apoptotic cells between control and mutant RGCs and not in necrosis because, in general, there is a very low percentage of necrotic cells. This could be due to the fact that the culture period is too short to observe a cell death analogous to that occurring in vivo when optic nerve damage occurs [43].

One of the main features of ADOA is a deficiency of oxidative phosphorylation, which has been extensively tested in fibroblasts from patients with different mutations in *OPA1* [44,45]. Therefore, the oxygen consumption of the generated RGCs was studied in order to monitor their bioenergetic function. Importantly, patient-derived RGCs showed a decrease in basal, ATP-dependent, and maximal respiration with respect to control RGCs, revealing a defect in mitochondrial function in the RGCs obtained from the ADOA plus patient. This is in line with the observations made by [32] Sladen et al. in 2022. This group modeled ADOA using iPSC lines with distinct pathogenic variants. Once they differentiated the lines into RGCs, they observed a reduction in their basal respiration compared to the corresponding controls, as well as reduced levels of ATP production. However, maximal respiration was shown to be diminished for the iPSC line with ADOA plus, harboring the missense variant c.1334G>A (p.Arg445His). Interestingly, the line presenting a nonsense variant, c.2708_2711delTTAG (p.Arg905Ter), that gave rise to isolated ADOA showed no decrease in this parameter. These observations, together with the results shown in this work, suggest a more severe impairment of mitochondrial function in syndromic ADOA, independent of the pathogenic variant causing the disease [32].

Additionally, the effect of the pathogenic variant c.1861C>T (p.Gln621Ter) on OPA1 expression at both the mRNA and protein levels was studied. These analyses showed that patient RGCs had an approximately 50% reduction in OPA1 in comparison with its matched isogenic control RGCs. When we correct the pathogenic variant using gene editing, the rescue of *OPA1* expression after differentiation towards RGCs has been achieved, backing up the suitability of the corrected iPSC line to be employed as an isogenic control. The 50% reduction in OPA1 expression detected in the created RGC model of ADOA supports the idea of haploinsufficiency being the main pathological mechanism in the disease for this patient [46,47], and it is probably indicative of reduced transcript stability due to the non-sense-mediated decay (NMD) mechanism. It has been shown that the majority of *OPA1* mutations giving rise to a premature stop codon provoke the initiation of NMD to prevent the accumulation of truncated peptides. The mRNA levels are reduced by 1.25- to 2.5-fold, and there are variations between different pathogenic variants, and even between individuals [48]. Future studies will be necessary to verify if this mechanism is really occurring in the case of the variant under study, for instance, by quantitative analysis using fluorescent reporters [49], in order to explain the decrease in the amount of protein observed in patient RGCs.

In summary, the generated isogenic RGC model of ADOA showed impaired mitochondrial function, an increase in apoptosis and ROS generation, diminished OPA1 expression, and mtDNA depletion, thus confirming that the generated model mimics the main biochemical features of ADOA.

Noticeably, the frequency with which phenotypes caused by ADOA plus occur highlights, in the context of the full spectrum of the disease, the impact of pathogenic mutations in *OPA1*, which may extend beyond involvement of the retina and the RGCs. Since RGCs are not the only cell type affected in ADOA plus, the availability of an iPSC line from a patient with the syndromic form of the disease will be very useful as it will allow its differentiation to other affected cell types, such as inner ear hair cells or Purkinje neuronal cells. In this way, new disease models would be generated in order to obtain a more global view of the pathophysiological mechanisms that produce the disease in the different tissues.

Recently, the likely pharmacological action of natural compounds and dietary supplements with antioxidant properties has been explored as a promising therapeutic alternative. Among them, nicotinamide, or vitamin B3, has shown potential for restoring *OPA1* expression in animal models [50]. In addition, in 2020, the first clinical trial with nicotinamide in glaucoma patients was successfully carried out [51]. In this article, we evaluated the potential of nicotinamide for the rescue of *OPA1* expression using as a platform the generated ADOA plus model as a platform. Remarkably, the treatment with nicotinamide at 10 mM for 24 h yielded a significant increase in *OPA1* expression levels in the patient RGCs, with a tendency to balance the values with the levels of control RGCs. Consequently, *OPA1* expression analysis by qPCR can be a valuable tool to be used as an easy read-out method to carry out future drug screening studies in the isogenic ADOA plus model. Further analyses will also be necessary to assess the potential of nicotinamide to rescue the biochemical alterations observed in this model.

In conclusion, the present study highlights the potential of the generated human RGC model for ADOA plus as a platform to search for personalized treatments against this disabling disorder.

## 4. Materials and Methods

### 4.1. iPSC Culture Conditions

iPSCs were maintained in mTeSR^TM^1 medium (StemCell Technologies, Vancouver, BC, Canada; #058520) on Matrigel hESC-qualified (Corning, New York, NY, USA; #354277) coated plates. Cells were dissociated and subcultured every 3–4 days with ReLeSR^TM^ (StemCell Technologies, Vancouver, BC, Canada; #05872) and cultured at 37 °C and 5% CO_2_.

### 4.2. Gene Editing with CRISPR/Cas9

#### 4.2.1. Design of the sgRNAs

The crRNA domain of two potential sgRNAs (named sgRNA.1 and sgRNA.2) targeting exon 20 of the *OPA1* gene was designed in order to correct the pathogenic variant in the iPSC line Oex20.4, previously generated in our laboratory. To this end, we employed the tool dCustom Alt-R™ CRISPR/Cas9 guide RNA (IDT, Newark, NJ, USA). We took into account for the design the proximity of the guide sequence to the pathogenic variant NM_015560.3: c.1861C>T (p.Gln621Ter), giving priority to those guides containing the mutation, which increases editing efficiency. It is also required to have a protospacer adjacent motif (PAM) adjacent to the guide sequence, that is, NGG for Cas9 from *Streptococcus pyogenes*. Data obtained from prediction tools were evaluated to choose those with higher on-target efficiencies and a lower likelihood of off-target modifications. The sequences of the crRNA domains that were selected are shown in Table 1.

The sgRNA complexes were formed by mixing the designed crRNA and the tracrRNA (IDT, Newark, NJ, USA; #1072532) at equimolar concentrations and annealing them at 95 °C for 2 min.

#### 4.2.2. Design of the ssODN

An ssODN was designed to be used as a template for repair via the HDR pathway after the double-strand break induced by Cas9. The desired modification to correct the pathogenic variant c.1861C>T (p.Gln621Ter) was included, along with two 90 nt-long homologous arms at both sides of the change. The ssODN sequence, which was ordered as an Ultramer oligonucleotide, is specified below. The modification is marked in bold, while the arms are shown underlined:


AATGATACTTCAGTCAAGCTGTTTTTAAAAACAATATTATATTTAGATTTGGTG

CTTTTGATACTTTTTTATTTCAGGGAGGAAATCCTT
**C**
AACAATCTTTGTGGGA

AAGAGTATCAACTCATGTGATTGAAAACATCTACCTTCCAGCTGCGCAGACCA

TGAATTCAGGAACTTTTAACA


#### 4.2.3. Gene Editing

Gene editing was performed as described by Bruntraeger et al. (2019) [21]. We started the procedure with the previously generated Oex2054SV.4 iPSC line (Oex20.4) [45]) grown in a 100 mm plate at 70% confluency and treated for 2 h with StemMACS™ Y27632 Rock inhibitor (Miltenyi Biotec, Bergisch Gladbach, Germany; #130-103-922). The protocol is based on the generation of RNP complexes with the sgRNA and the high-fidelity Cas9 Alt-R^®^ S.p. HiFi Cas9 Nuclease V3 (IDT, Newark, NJ, USA; #1081061) in combination with a ssODN as repair template for the induced modification. The nucleofection was performed with the P3 Primary Cell 4D-Nucleofector™ X Kit (Lonza, Basel, Switzerland; #PBP3-02250) in a 4D Nucleofector System by Lonza (Program CA-137), following the instructions of the manufacturer. Afterwards, potentially edited iPSCs were further cultured in mTeSR^TM^1.

#### 4.2.4. T7E1 Assay to Evaluate Cutting Efficiency

An assay with the T7 endonuclease was performed in order to evaluate the Cas9 cutting efficiency in combination with each sgRNA. iPSCs were nucleofected with RNPs formed by the Cas9 nuclease in combination with sgRNA.1 or sgRNA.2 without ssODN addition. Then, genomic DNA was extracted from the pool of nucleofected iPSCs using the kit NucleoSpin^®^ Tissue (Macherey-Nagel, Düren, Germany; #740952). KAPA HiFi HotStart (Roche, Basel, Switzerland; #7958935001) was used to amplify the region of interest for 35 cycles with the primers OPA1-Fw (5′-ATGCGTTCATTATCTTGACTGG-3′) and OPA1-Rv (5′-CTCTATAATCCACAGAATCCAC-3′). The PCR product was purified with the kit NZYGelpure (NZYtech, Lisbon, Portugal; #MB01102). Subsequently, 200 ng of the product were used to perform the heteroduplex formation and then were digested with the T7E1 enzyme (New England Biolabs, Ipswich, MA, USA; #M0302S) for 15 min at 37 °C. The digested product was loaded in a 2% agarose gel, and the percentage of gene modification was estimated by calculating band intensities with Image J (NIH) software (version 1.54f) and applying the following equation: $\%\;Modification=100\times \left(1-\sqrt{\left(1-F_{cut}\right)}\right)$ where *F_cut_* is the fraction digested, corresponding to the sum of the intensities of the cleaved bands divided by the sum of total band intensities [52].

#### 4.2.5. Evaluation of Editing Efficiency with RFLP

After the nucleofection of iPSCs with the RNPs formed by Cas9 and sgRNA.1 and the addition of ssODN, we performed a Restriction Fragment Length Polymorphism (RFLP) to evaluate the editing efficiency. For this purpose, genomic DNA was extracted using the NucleoSpin^®^ Tissue kit (Macherey-Nagel, Düren, Germany; #740952) and amplified with the primers OPA1-Fw (5′-ATGCGTT CATTATCTTGACTGG-3′) and OPA1-Rv (5′-CACATTCAAGCAAATACT CAAGC-3′) and purified, as described in the previous section. Afterwards, 500 ng of the product were digested with the enzyme XmnI (New England Biolabs, Ipswich, MA, USA; #R0194L) following the manufacturer’s instructions. The digested product was loaded on a 2% agarose gel, and the percentage of editing was calculated by quantifying the band intensities of the cleaved fractions with the ImageJ NIH software (version 1.54f).

#### 4.2.6. Subcloning

When the edited iPSC pool reached 70% confluency, it was detached with StemPro^TM^ Accutase^TM^ (Gibco, Waltham, MA, USA; #A1110501) to create a single-cell suspension. Subsequently, 1000 cells were seeded on hESC-qualified Matrigel-coated 10 cm plates with cloning medium composed of mTeSR^TM^1 and 10% CloneR^TM^ (StemCell Technologies, Vancouver, BC, Canada; #05888). Plates were incubated for two days when the cloning medium was changed, and on the third day, 25% of the cloning medium was added. The next day, medium was renovated with mTeSR^TM^1 until colonies reached approximately 1–2 mm in diameter.

#### 4.2.7. Freezing and Analysis of the Clones

We manually picked 94 colonies that come from a single cell. These were picked in two 96-well hESC-qualified Matrigel-coated plates to create a replicate. When the colonies reached an appropriate size, we froze the colonies on one plate using Knockout^TM^ serum (Gibco, Waltham, MA, USA; #11520366) with 10% final DMSO. Colonies from the other plate were lysated with Yolk Sac lysis buffer (Tris–HCl 10 mm pH 8.3, KCl 50 mm, MgCl_2_ 2 mm, IGEPAL CA-630 0.45%, and TWEEN^®^20 0.45%) supplemented with proteinase K (GE Healthcare, Chicago, IL, USA; #406172) at 60 °C for 1 h. Subsequently, proteinase K was inactivated at 95 °C for 10 min. A 1/10 dilution of the lysates was amplified by PCR as described in Section 4.2.5, and the PCR products were purified using the MultiScreen PCR_μ96_ filtration plates (Millipore, Burlington, MA, USA; #LSKMPCR10). Afterwards, an RFLP analysis was performed for each clone as described in Section 4.2.5, and the positive ones were double-checked by Sanger sequencing.

### 4.3. Pluripotency and Integrity Assessment

#### 4.3.1. qPCR

RNA extraction was carried out with TRI Reagent^TM^ Solution (Invitrogen, Waltham, MA, USA; #AM9738). Then, the retrotranscription of 1 µg of the extracted RNA was performed using the RevertAid RT kit (Thermo Fisher Scientific, Waltham, MA, USA; #K1691). We performed the qPCR amplification in a 7500 Fast Real-Time PCR System by Applied Biosystems (Waltham, MA, USA) using the GoTaq^®^ qPCR Master Mix (Promega, Madison, WI, USA; #A6002) following the manufacturer’s instructions. Expression levels were normalized to the housekeeping gene GAPDH, and three independent replicates were performed. The sequence of the primers used can be found in Cerrada et al., 2020 [22].

#### 4.3.2. Immunocytochemistry Analyses

iPSCs were fixed with a 10% formalin solution (Merck, Darmstadt, Germany; #HT501128) for 30 min at room temperature (RT). Then, cells were permeabilized using 0.1% Triton^TM^ X-100 (Merck, Darmstadt, Germany; #T8787) in Tris-Buffered Saline buffer (TBS) for 45 min at RT. Afterwards, blocking was performed with 3% donkey serum (Merck, Darmstadt, Germany; #D9663) and 0.3% Triton^TM^ X-100 in TBS for 2 h at RT. Primary antibodies were incubated overnight at 4 °C and secondary antibodies for 2 h at RT in the dark (Table 2). Nuclei were stained with DAPI (Merck, Darmstadt, Germany; #28718-90-3).

#### 4.3.3. In Vitro Differentiation

For embryoid body generation and their in vitro spontaneous differentiation towards the three germ layers, the protocol described by Galera-Monge et al. (2019) was followed [53]. The expression of the markers α-smooth muscle actin (SMA) for mesoderm, α-fetoprotein (AFP) for endoderm, and class III β-tubulin (Tuj1) for ectoderm was assessed by immunocytochemistry, as described in Section 4.3.2. The antibodies used are listed in Table 3.

#### 4.3.4. STR Analysis

A DNA fingerprinting analysis was carried out to confirm that the edited clone came from the starting line Oex20.4 [20]. To this end, the subsequent markers were amplified by PCR, followed by a fragment analysis: D13S317, D7S820, VWA, D8S1179, D21S11, D19S433, D2S1338, and amelogenin for sex determination. The analysis was performed using the PeakScanner v3.5 software (Applied Biosystems, Waltham, MA, USA). The sequence of the PCR primers can be found in Ortuño-Costela et al., 2017 [54].

#### 4.3.5. Mycoplasma Detection

To evaluate whether the iPSCs were free of mycoplasma contamination, the supernatant of a 3-day confluent culture was boiled at 95 °C for 5 min and spun at 13,000 rpm for 5 s. We performed PCR amplification in a Verity Thermal Cycler (Applied Biosystems, Waltham, MA, USA) using the primers MGSO-Fw (5′-TGCACCATCTGTCACTCT GTTAACCTC-3′) and GPO-Rv (5′-GGGAGCAAACAGGATTAGATACCCT-3′). The PCR product was run on a 1.2% agarose gel. The band at 300 bp represents that the sample is positive for mycoplasma.

#### 4.3.6. Karyotype Analysis

iPSCs with more than twenty passages were treated with 10 µg/mL Colcemid™ (Gibco, Waltham, MA, USA; #15212012) at 37 °C for 90 min. Then, cells were trypsinized, treated with a 0.075 M KCl hypotonic solution, and fixed with Carnoy’s solution. Cells were dropped on a microscope glass slide. Wright staining was used for G-banding, with at least 20 metaphases analyzed. Digital images were acquired with a monochrome CCD camera linked to Metasystem software.

### 4.4. iPSC Differentiation towards RGCs

iPSC differentiation towards RGCs was performed following the procedure published by Lee et al. [23] (2018) with minor modifications. After reaching an approximate confluence of 70–80%, iPSCs growing with mTeSR^TM^1 medium on Matrigel-coated dishes were detached with StemPro^TM^ Accutase^TM^ (Gibco, Waltham, MA, USA; #A1110501) to form EBs. For this purpose, AggreWell^TM^800 plates (StemCell Technologies, Vancouver, BC, Canada; #34815) and Anti-Adherence Rinsing Solution (StemCell Technologies, Vancouver, BC, Canada; #07010) were used following the manufacturer’s instructions. EBs were created in EB medium (the composition of this medium is detailed in Lee et al., 2018) supplemented with 10 µM StemMACS^TM^ Y27632 Rock inhibitor (Miltenyi Biotec, Bergisch Gladbach, Germany; #130-103-922). At day 4, EBs were transferred to Matrigel-coated dishes and cultured with N2 medium (detailed composition in Table 4); at day 8, neural rosettes were completely formed in the center of the attached EBs and were selected with the STEMdiff^TM^ Neural Rosette Selection Reagent (StemCell Technologies, Vancouver, BC, Canada; #5832) following the manufacturer’s instructions. After gentle trituration, rosette clumps were plated on Matrigel Growth Factor Reduced-coated dishes (Corning, New York, NY, USA; #354230) with N2B27 medium (detailed composition in Table 5). RPCs grew from the clumps forming a monolayer, were cultured in the conditions described above, and were passed every 3–4 days with StemPro^TM^ Accutase^TM^ (Gibco, Waltham, MA, USA; #A1110501). For differentiation towards RGCs, RPCs were seeded onto poly-D-lysine (Merck, Darmstadt, Germany; #P1024) and laminin-coated dishes (Merck, Darmstadt, Germany; #L2020) in N2B27 medium supplemented with 4 µM DAPT (Merck, Darmstadt, Germany; #D5942). On the fourth day, 50 ng/mL BDNF (Miltenyi Biotec, Bergisch Gladbach, Germany; #130-096-286) was added, and DAPT was removed on day ten. RGCs were obtained after 28 days of differentiation and were used for further experiments.

### 4.5. Characterization of the Generated RPCs and RGCs

#### 4.5.1. RT-qPCR Analyses

The qPCR amplification was carried out as described in Section 4.3.1. The sequences of the primers are listed in Table 6. All the values are representative of at least three independent replicates, and the expression levels were normalized to the housekeeping gene *GAPDH*.

#### 4.5.2. Immunocytochemistry Analyses

For immunocytochemistry analyses, fixation, permeabilization, and blocking steps were performed as described in 4.3.2. Incubation with primary antibodies was carried out for 1.5 h at RT and with secondary antibodies at RT for 2 h in the dark (Table 7). Nuclei were stained with DAPI (Merck, Darmstadt, Germany; #28718-90-3).

### 4.6. Validation and Functional Analyses of the RGC Model

#### 4.6.1. OPA1 Expression Analysis by RT-PCR

For the expression analysis of *OPA1*, a qPCR amplification was performed as described in Section 4.3.1. The sequence of the oligonucleotides is listed in Table 8. All the values are representative of at least three independent experiments, and the expression levels were normalized to the housekeeping gene *GAPDH*.

#### 4.6.2. Western Blot for OPA1 Analysis

On day 28 of differentiation, pellets from RGCs were collected, and proteins were extracted using RIPA buffer (50 mm Tris-HCl pH 8, 150 mm NaCl, 1% Triton X-100, 0.1% SDS (NZYtech, Lisbon, Portugal; #151-21-3) and protease inhibitors (Roche, Basel, Switzerland; #4693132001) according to manufacturer specifications. Protein concentration was determined using the Quick Start Bradford 1X Dye Reagent (Bio-Rad, Hercules, CA, USA; #5000205) according to the manufacturer’s instructions. Then, we performed a SDS-PAGE in 7.5% resolving and 4% stacking acrylamide gels (Bio-Rad, Hercules, CA, USA; #1610156) and transferred the proteins to a nitrocellulose membrane (Bio-Rad, Hercules, CA, USA; #1704158) with a Mini-PROTEAN 3 Cell Transfer system (Bio-Rad, Hercules, CA, USA; #165-3301) at 4 °C for 90 min at 110 V. The blocking of the membrane was performed with 5% non-fat dry milk and 0.1% Tween-20 (Merck, Darmstadt, Germany; #P1379) in PBS for 2 h at RT. The OPA1 primary antibody (Invitrogen, Waltham, MA, USA; #MA5-16149) was diluted 1:1000 in 1% non-fat dry milk and 0.1% Tween-20 in PBS, and the incubation was overnight at 4 °C with gentle agitation. HRP secondary antibody (Invitrogen, Waltham, MA, USA; #A16072) was diluted 1:5000 in 0.1% Tween-20 in PBS, and it was incubated at RT for 1 h. Actin primary antibody (Invitrogen, Waltham, MA, USA; #MA5-11869) at 1:3500 with the HRP secondary antibody (1:40,000) was used as a loading control. Then, the membrane was revealed with Clarity Max™ Western ECL Blotting Substrates (Bio-Rad, Hercules, CA, USA; #1705062). Protein band quantification was performed using the software Image Lab 5.0 (Bio-Rad, Hercules, CA, USA).

#### 4.6.3. Identification of mtDNA Deletions by Long-Range PCR

We performed a long-range PCR to evaluate the presence of mtDNA deletions using 100 ng of total DNA and following the protocol described by Bonneau et al. (2008) [55]. For this purpose, the DNA polymerase Takara LA Taq (Takara Bio, San Jose, CA, USA; #RR002A) was employed. The sequences of the primers are shown in Table 9. The amplification product was loaded on a 0.8% agarose gel, resulting in a 7315 bp product in the absence of multiple deletions.

#### 4.6.4. qPCR Relative Quantification of mtDNA

Relative mtDNA copy number was determined through simultaneous assessment of the nuclear gene *RNAse P* and the mitochondrial gene *MT-RNR1* using qPCR and as starting material 25 ng of total DNA. qPCR amplification was performed in a 7500 Fast Real-Time PCR System by Applied Biosystems (Waltham, MA, USA) using the GoTaq^®^ qPCR Master Mix (Promega, Madison, WI, USA; #A6002) following the manufacturer’s instructions. Primers used are listed in Table 10. All the values are representative of at least three independent replicates.

#### 4.6.5. Analysis of Mitochondrial Respiration

A Seahorse XFp Extracellular Flux Analyzer (Seahorse Biosciences, North Billerica, MA, USA) was used to measure the rates of oxygen consumption. RGCs at day 25 of differentiation generated from RPCs were detached with StemPro^TM^ Accutase^TM^ (Gibco, Waltham, MA, USA; #A1110501) for 30 min at 37 °C. Afterwards, RGCs were partially dissociated and filtered to discard single cells. RGC clusters were plated on poly-D-lysine and laminin-coated Seahorse microplates with N2B27 medium supplemented with BDNF (Miltenyi Biotec, Bergisch Gladbach, Germany; #130-096-286) and cultured for 3 days more. The day before performing the experiment, the XFp cartridge was equilibrated with the calibration buffer overnight at 37 °C. Seahorse XFp medium was supplemented with 1 mm of pyruvate, 2 mm of glutamine, and 10 mm of glucose just before performing the experiment (Agilent Technologies, Santa Clara, CA, USA; #103680-100). The Mito Stress Test Kit compounds (Agilent Technologies, Santa Clara, CA, USA; #103010-100) were loaded in the ports following the manufacturer’s instructions at final concentrations of 1.5 µM for oligomycin, 1 µM for FCCP, and 0.5 µM for Rotenone/Antimicin A. The optimal uncoupling concentration of FCCP was determined after previous titration. All the values are representative of at least three independent replicates and were normalized to the total protein quantity using the Quick Start Bradford 1X Dye Reagent (Bio-Rad, Hercules, CA, USA; #5000205).

#### 4.6.6. Quantification of Reactive Oxygen Species (ROS)

The detection of ROS species was performed using the DCFH-DA probe (Thermo Fisher Scientific, Waltham, MA, USA; # C2938). Oex20.4 and their matched edited iPSC-derived RGCs between days 28 and 32 of differentiation were incubated with 1 µM DCFH-DA for 10 min at 37 °C. Then, cells were washed twice with PBS 1X and detached with TrypLE^TM^ Express (Gibco, Waltham, MA, USA; #12605010). Cells were partially triturated, centrifuged at 200× *g* for 5 min at 4 °C, and suspended in cold PBS 1X. After that, cells were filtered with a 100 µm strainer to ensure a single cell suspension, and 1 × 10^6^ cells were aliquoted for each condition. The accumulation of DCF in the cells was measured by an increase in fluorescence at 530 nm when the sample was excited at 485 nm with a FACSCalibur flow cytometer (Beckton Dickinson, Franklin Lakes, NJ, USA). The collected data was analyzed with the software FlowJo V10 (BD Biosciences, Franklin Lakes, NJ, USA).

#### 4.6.7. Apoptosis Quantification

Apoptosis detection was performed using the Annexin V Apoptosis Detection Kit with propidium iodide (PI) (Immunostep, Salamanca, Spain; #ANXVKDY-100T). Oex20.4 and Oex20.4-B3 iPSC-derived RGCs between days 28–32 of differentiation were detached with TrypLE^TM^ Express. Cells were partially triturated, spun at 200× *g* for 5 min at 4 °C, and filtered in binding buffer with a 100 µm strainer. For each condition, 1 × 10^6^ cells were aliquoted and stained with 1 µL of Annexin V and 1 µL of PI for 7 min. The fluorescence increase at 530 nm was determined when cells were excited at 485 nm with a FACSCalibur flow cytometer (Beckton Dickinson, Franklin Lakes, NJ, USA). The collected data were analyzed with the software FlowJo V10 (BD Biosciences, Franklin Lakes, NJ, USA).

### 4.7. Analysis of the Nicotinamide Potential for the Rescue of OPA1 Expression

We performed a pilot study in order to evaluate the potential of nicotinamide for the rescue of *OPA1* expression in the patient’s RGCs. To this end, Oex20.4 and its matched isogenic control were differentiated towards RGCs as described in Section 4.4 and characterized following the protocols detailed in Section 4.5. Once RGCs were generated, we treated them with 10 mM nicotinamide (StemCell Technologies, Vancouver, BC, Canada; #07154) for 24 and 48 h and 25 mM for 24 h. After the treatment, cell pellets were collected, and a RT-qPCR analysis was carried out as described in Section 4.6.1, using *GAPDH* as a housekeeping gene.

## Figures and Tables

**Table 1:** Sequence of crRNA domains designed with the IDT tool, including on-target and off-target scores. MM: mismatches.

Strand	crRNA Sequence (5′→3′)	PAM	On-Target Score	Off-Target Score	Off-Targets
+	AATCCTTTAACAATCTTTGT	GGG	75	0	1 (3–4 MM)
−	TTTCCCACAAAGATTGTTAA	AGG	79	0	3 (3–4 MM)

**Table 2:** Primary and secondary antibodies used to detect the expression of the pluripotency markers by immunocytochemistry.

**Primary Antibodies**		
**Antibody**	**Dilution**	**Reference**
Mouse Anti-TRA-1-81	1:150	Millipore, Burlington, MA, USA; #MAB4381
Mouse Anti-TRA-1-60	1:150	Millipore, Burlington, MA, USA; #MAB4360
Rabbit Anti-SOX2	1:100	Thermo Fisher Scientific, Waltham, MA, USA; #PA1-16968
Mouse Anti-SSEA4	1:10	Millipore, Burlington, MA, USA; #MAB4304
Rat Anti-SSEA3	1:20	Abcam, Cambridge, UK; #ab16286
Goat Anti-NANOG	1:25	R&D Systems, Minneapolis, MN, USA; #sc-5279
Mouse Anti-OCT4	1:100	Santa Cruz Biotechnology, Dallas, TX, USA; #sc-5279
**Secondary antibodies**		
**Antibody**	**Dilution**	**Reference**
Cy^TM^2-conjugated AffiniPure Donkey Anti-Goat IgG (H + L)	1:50	Jackson ImmunoResearch Labs, Ely, UK; #705-225-147
Cy^TM^2-conjugated AffiniPure Goat Anti-Mouse IgG, Fcγ Subclass 2b specific	1:50	Jackson ImmunoResearch Labs, Ely, UK; #115-225-207
Cy^TM^2-conjugated AffiniPure anti-conejo IgG (H + L)	1:50	Jackson ImmunoResearch Labs, Ely, UK; #111-225-144
Cy^TM^3-conjugated AffiniPure Goat Anti-Rat IgM, μ chain specific	1:250	Jackson ImmunoResearch Labs, Ely, UK; #112-165-075
Cy^TM^-3conjugated AffiniPure Goat Anti-Mouse IgG, Fcγ Subclass 3 specific	1:250	Jackson ImmunoResearch Labs, Ely, UK; #115-165-209
Cy^TM^-3conjugated AffiniPure Donkey Anti-Mouse IgM, μ chain specific	1:250	Jackson ImmunoResearch Labs, Ely, UK; #115-165-020
Alexa Fluor 488, Goat Anti-Mouse IgG (H + L)	1:500	Thermo Fisher Scientific, Waltham, MA, USA; #A32723
Alexa fluor 568, Goat Anti-Rabbit IgG (H + L)	1:800	Thermo Fisher Scientific, Waltham, MA, USA; #A-11011

**Table 3:** Primary and secondary antibodies used to analyze by immunocytochemistry the expression of the markers for ectoderm, mesoderm, and endoderm after in vitro differentiation.

**Primary Antibodies**		
**Antibody**	**Dilution**	**Reference**
Mouse Anti-AFP (endoderm)	1:300	Merck, Darmstadt, Germany; #WH0000174M1
Mouse Anti-SMA (mesoderm)	1:400	Merck, Darmstadt, Germany; #A2547
Mouse Anti-Tuj1 (ectoderm)	1:300	Merck, Darmstadt, Germany; #T8660
**Secondary antibodies**		
**Antibody**	**Dilution**	**Reference**
Goat Anti-Mouse Alexa Fluor^®^ 488	1:500	Thermo Fisher Scientific, Waltham, MA, USA; #A-11029

**Table 4:** N2 medium composition for the differentiation towards RGCs [23].

N2 Medium			
Composition	Concentration	Supplier	Reference
DMEM/F-12	-	Gibco	11320-033
N2 supplement (100X)	1X	Gibco	17504044
Insulin solution	20 mg/mL	Sigma	I9278
XAV939	1 µM	StemCell Technologies	72672
Human FGF-2	60 ng/mL	Miltenyi Biotec	130-093-838

**Table 5:** N2B27 medium composition for the differentiation towards RGCs [23].

N2B27 Medium			
Composition	Concentration	Supplier	Reference
DMEM/F-12	-	Gibco	11320-033
N2 supplement (100X)	1X	Gibco	17504044
B27 supplement without vitamin A (50X)	1X	Gibco	12587-001
Human FGF-2	20 ng/mL	Miltenyi Biotec	130-093-838

**Table 6:** Sequence of the primers employed to assess the expression of typical RPC and RGC markers by qPCR.

Primer	Sequence (5′-3′)
PAX6-Fw	CAGATGTGTTTGCCCGAGAA
PAX6-Rv	GCCTGTCTTCTCTGATTCCT
RX-Fw	CAAGGTCAACCTACCAGAGGT
RX-Rv	CAGCTTCATGGAGGACACTTC
CHX10-Fw	CGGAGTATGGGCTCTACGG
CHX10-Rv	CTCCATCTTGTCGAGCTTGG
SIX6-Fw	TACGCAGGTGGGCAACTGGT
SIX6-Rv	GTAGTGCCCGCCCGGAAC
BRN3A-Fw	CTCCCTGAGCACAAGTACCC
BRN3A-Rv	CCGGCTTGAAAGGATGGCTC
BRN3B-Fw	CTCGGAGGCTATGCGGAGA
BRN3B-Rv	TGGTAGGTGGCGTCCGGTT
ISLET1-Fw	AAGGACAAGAAGCGAAGCAT
ISLET1-Rv	TTCCTGTCATCCCCTGGATA
SNCG-Fw	TGGTGAGCAGCGTCAACACT
SNCG-Rv	CCTCTTTCTCTTTGGATGCC
ATOH7-Fw	GTCTCCACTGTGAGCACTTC
ATOH7-Rv	GAAGCCGAAGAGTCTCTGGC
THY1-Fw	AGGTCCTCTACTTATCCGCC
THY1-Rv	ATGCCCTCACACTTGACCAG
GAPDH-Fw	GCACCGTCAAGGCTGAGAAC
GAPDH-Rv	AGGGATCTCGCTCCTGGAA

**Table 7:** Primary and secondary antibodies were used to analyze by immunocytochemistry the expression of the typical RGC markers.

**Primary Antibodies**		
**Antibody**	**Dilution**	**Reference**
Rabbit anti-PAX6	1:500	Thermo Fisher Scientific, Waltham, MA, USA; #PA5-85374
Mouse anti-BRN3A	1:50	Santa Cruz Biotechnology, Dallas, TX, USA; #sc-8429
Rabbit anti-SNCG	1:500	Thermo Fisher Scientific, Waltham, MA, USA; #PA5-29142
Mouse anti-THY1	1:100	Thermo Fisher Scientific, Waltham, MA, USA; # MA5-16671
Mouse anti-TUJ1	1:300	Merck, Darmstadt, Germany; #T8660
**Secondary antibodies**		
**Antibody**	**Dilution**	**Reference**
Alexa Fluor 488, Goat Anti-Mouse IgG (H + L)	1:500	Thermo Fisher Scientific, Waltham, MA, USA; #A32723
Alexa fluor 568, Goat anti-Rabbit IgG (H + L)	1:800	Thermo Fisher Scientific, Waltham, MA, USA; #A-11011

**Table 8:** Sequence of the primers used to evaluate *OPA1* expression in the generated RGCs.

Primer	Sequence (5′-3′)
OPA1-Fw	CAGATGTGTTTGCCCGAGAA
OPA1-Rv	GCCTGTCTTCTCTGATTCCT
GAPDH-Fw	GCACCGTCAAGGCTGAGAAC
GAPDH-Rv	AGGGATCTCGCTCCTGGAA

**Table 9:** Sequence of the primers employed to detect mtDNA deletions by long-range PCR.

Primer	Sequence (5′-3′)
F8285-8314 (FW)	CTCTAGAGCCCACTGTAAAGCTAACTTAGC
R15600-15574 (RV)	GGGACGGATCGGAGAATTGTGTAGGCG

**Table 10:** Sequence of the primers used to analyze mtDNA depletion.

Primer	Sequence (5′-3′)
12S-FW	CCACGGGAAACAGCAGTGAT
12S-RV	CTATTGACTTGGGTTAATCGTGTGA
ARNase P-FW	GCTCTCTGAAAGTGACGCC
ARNase P-RV	CTCCATGGAGAAGCGCTGC

## Data Availability

The original contributions presented in the study are included in the article, further inquiries can be directed to the corresponding author.

## Supplementary Materials

The supporting information can be downloaded at: https://www.mdpi.com/article/10.3390/ijms25137240/s1, reference [56] is cited in Supplementary Materials.
