# Supplementary material for: Creation of an Isogenic Human iPSC-Based RGC Model of Dominant Optic Atrophy Harboring the Pathogenic Variant c.1861C>T (p.Gln621Ter) in the OPA1 Gene
Source: Int J Mol Sci. 2024 Jun 30;25(13):7240. doi: 10.3390/ijms25137240 (PMC11242102; doi:10.3390/ijms25137240)
Supplement: Supplementary file 1 [file ijms-25-07240-s001.zip › ijms-3020901-supplementary.pdf]

Supplementary Figures

Creation of an isogenic human iPSC-based RGC model of dominant optic atrophy harbouring the pathogenic variant c.1861C>T (p.Gln621Ter) in the *OPA1* gene

Marta García-López, Lydia Jiménez-Vicente, Raquel González-Jabardo, Helena Dorado, Irene Gómez-Manjón, Miguel Ángel Martín, Carmen Ayuso, Joaquín Arenas, and María Esther Gallardo

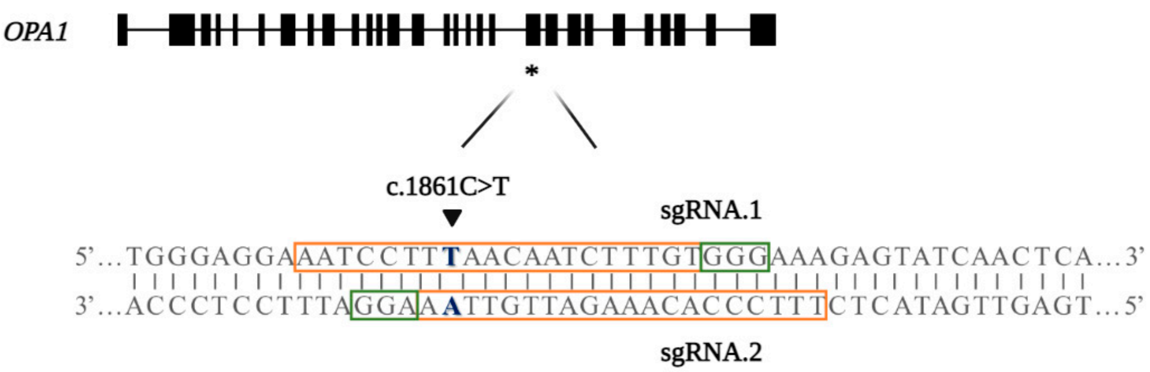

Figure S1. General scheme of the gene editing strategy. Representation of the localization of the two designed sgRNAs targeting exon 20 of the *OPA1* gene (indicated with an asterisk). Both RNA guides, with high on-target efficiency and low off-target activities, contain the pathogenic variant NM_015560.3: c.1861C>T (p.Gln621Ter) on their sequence (marked in blue). sgRNA sequences are marked in orange, while PAM sequences are marked in green.

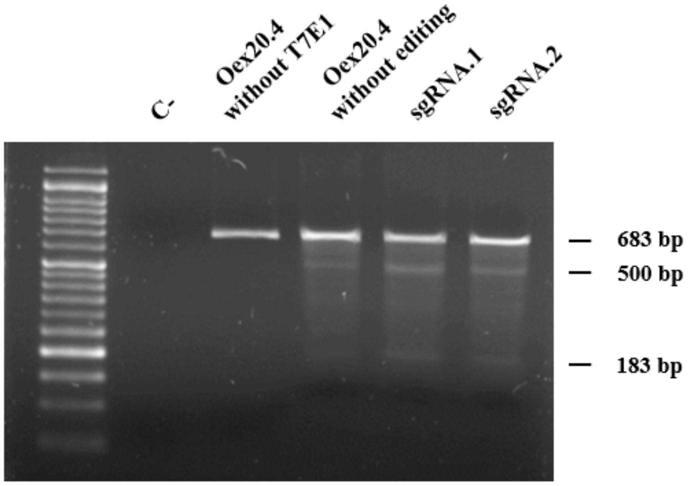

Figure S2. Endonuclease T7E1 assay to evaluate the efficiency of the designed sgRNAs. The cutting efficiency was calculated for both sgRNA.1 (12%) and sgRNA.2 (7%) by comparing the band intensities of the digested fractions. Oex20.4 without T7E1: control without T7 endonuclease; Oex20.4 without editing: control of T7E1 cutting in the non-nucleofected cell pool; sgRNA.1 and sgRNA.2: Oex20.4 iPSCs nucleofected with each RNA guide and after cutting with T7 endonuclease.

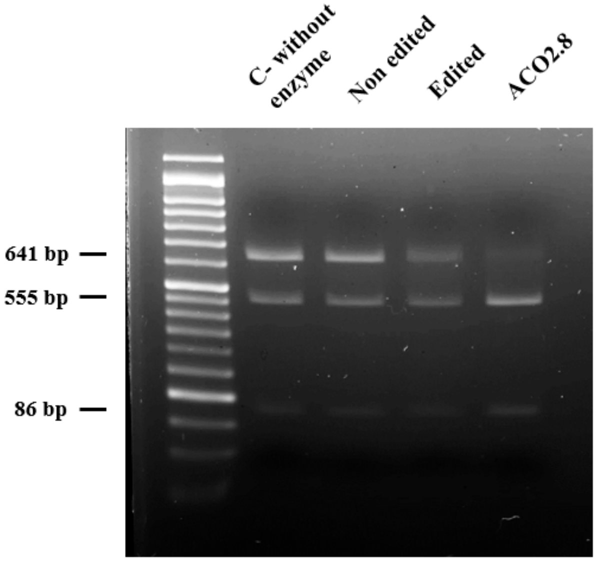

Figure S3. RFLP analysis to evaluate the editing efficiency. The editing percentage (15%) was calculated by comparing the band intensities of the digested fractions after the digestion with *XmnI* of the non-edited cells (30%) and edited cells (45%). C- without enzyme: edited iPSC pool without *XmnI* enzyme; non-edited: no nucleofected iPSCs; edited: iPSCs nucleofected with RNPs formed by Cas9 and sgRNA.1 with ssODN; ACO2.8: another iPSC line without the pathogenic variant to be edited [1] (positive control for the digestion with *XmnI*).

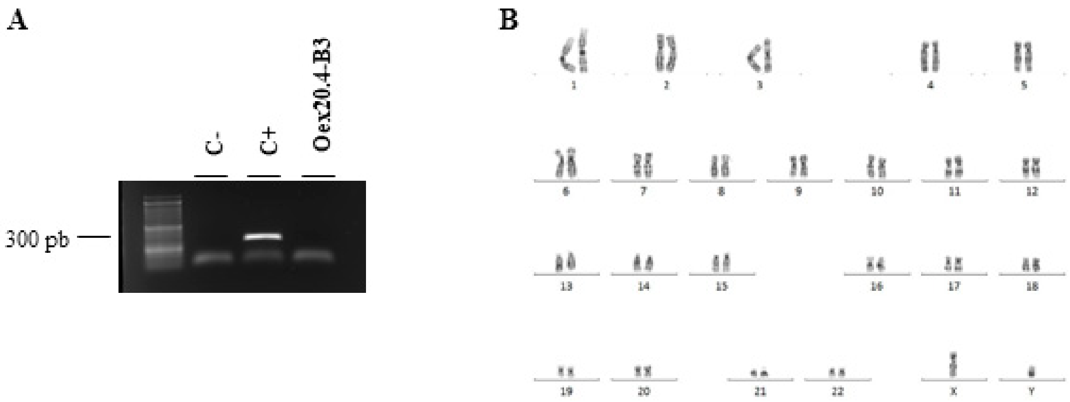

Figure S4. Integrity assessment of the iPSC line Oex20.4-B3. (A) Mycoplasma analysis showing that the edited line Oex20.4-B3 is mycoplasma free, as indicated by the absence of a band at 300 bp. C-: negative control, H_2O; C+: positive control, mycoplasma-positive sample. (B) Normal 46, XY karyotype of the edited iPSC line Oex20.4-B3.

Reference:

1. Cerrada, V.; García-López, M.; Moreno-Izquierdo, A.; Villaverde, C.; Zurita, O.; Martin-Merida, M.I.; Arenas, J.; Ayuso, C.; Gallardo, M.E. Derivation of a human DOA iPSC line, IISHDOi006-A, with a mutation in the ACO2 gene: c.1999G>A; p.Glu667Lys. *Stem Cell Res.* **2019**, *40*, 101566, doi:10.1016/j.scr.2019.101566.
